# Estimation of the Lethality Rate, Recovery Rate, and Case Fatality Ratio of Classical Swine Fever in Japanese Wild Boar: An Analysis of the Epidemics From September 2018 to March 2019

**DOI:** 10.3389/fvets.2021.772995

**Published:** 2021-12-15

**Authors:** Ryota Matsuyama, Takehisa Yamamoto, Yoko Hayama, Ryosuke Omori

**Affiliations:** ^1^Graduate School of Biomedical and Health Sciences, Hiroshima University, Hiroshima, Japan; ^2^Epidemiology Research Unit, Viral Disease and Epidemiology Research Division, National Institute of Animal Health, National Agriculture and Food Research Organization, Tsukuba, Japan; ^3^International Institute for Zoonosis Control, Hokkaido University, Sapporo, Japan

**Keywords:** classical swine fever, case fatality ratio, mathematical model, wild boar, wild animal, communicable disease, wildlife disease

## Abstract

Understanding the morbidity and lethality of diseases is necessary to evaluate the effectiveness of countermeasure against the epidemics (e.g., vaccination). To estimate them, detailed data on host population dynamics are required; however, estimating the population size for wildlife is often difficult. We aimed to elucidate the morbidity and lethality of classical swine fever (CSF) currently highly prevalent in the wild boar population in Japan. To this end, we estimated lethality rate, recovery rate, and case fatality ratio (CFR) of CSF without detailed data on the population estimates of wild boar. A mathematical model was constructed to describe the CSF dynamics and population dynamics of wild boar. We fitted the model to the (i) results of the reverse transcription polymerase chain reaction (RT-PCR) test for the CSFV gene and the (ii) results of the enzyme-linked immunosorbent assay (ELISA) test for the antibody against CSFV in sampled wild boar. In the 280 wild boar sampled from September 2018 to March 2019 in the major CSF-affected area in Japan, the lethality rate and recovery rate of CSF per week were estimated as 0.165 (95% confidence interval: 0.081–0.250) and 0.004 (0–0.009), respectively. While the estimate of lethality rate of CSF was similar with the estimates in previous studies, the recovery rate was lower than those reported previously. CFR was estimated as 0.959 (0.904–0.981) using our estimate of recovery rate. This study is the first to estimate lethality rate of CSF from the dynamics of CSF epidemics in the wild boar population. Since the value of CFR is sensitive to the value of recovery rate, the accuracy in the estimate of recovery rate is a key for the accurate estimation of CFR. A long-term transmission experiment of moderately virulent strains may lead to more accurate estimation of the recovery rate and CFR of CSF.

## Introduction

Classical swine fever (CSF) is one of the most important infectious diseases in the pig farming industry because of its significant impact on animal health and economic losses (1–3). The disease is caused by the CSF virus (CSFV), which belongs to the genus *Pestivirus* of the Flaviviridae family, and its obligate host is the family *Suidae* (e.g., domesticated pigs and Eurasian wild boar (*Sus scrofa*)) (3, 4). The introduction of CSF to susceptible populations can sometimes result in substantial morbidity and mortality. Therefore, it is designated as a notifiable disease by the World Organisation for Animal Health (5).

Understanding both morbidity and lethality is essential for controlling the CSF epidemics. The course of morbidity and mortality events varies with the virulence of the CSFV strains, age of the hosts, breeds (in pigs), and the living environment (domestic or wild animals) (2, 6). The clinical course of CSF has been classified into three forms according to the duration of clinical phases: acute, chronic, and persistent (7). Often, high mortality is seen in the acute form, especially in piglets with fever (usually over 40°C), diarrhoea, and neurological signs (7). When the duration of the clinical phase is longer than the acute form (e.g., 2–4 weeks), the form is called sub-acute (8). The chronic form causes various non-specific signs, including fever, loss of appetite, and death 2–3 months after infection. The persistent form occurs in piglets infected with CSF through vertical transmission, resulting in poor growth and death several months after birth (3, 7). Post-natal persistent infection with moderately virulent CSFV strains has also been reported in piglets between their birth and 3 weeks of age (3, 9). Particularly, infections with highly virulent strains in vulnerable hosts may cause high mortality (2, 10), and the case fatality ratio (CFR) among young and adult domestic pigs may reach 80–100% (11). CFR is equivalent to [the number of death by infection]/[the number of infected individuals]. Wild boar and feral pigs are believed to demonstrate clinical reactions similar to those of domestic pigs (2). The actual proportion of CSF-induced death among infected individuals in wild boar, i.e. [the number of death by CSFV infection]/[the number of CSFV infected wild boar], is unclear due to the difficulty of observation, and it has been believed to reach 90% (2). Considering that the experimental infection of moderately virulent CSFV (genotype 2.3) resulted in only the sub-clinical course of CSF infection in 8 month-old wild boar (12), lethality lower than 90% may occur in field conditions. However, the actual morbidity and lethality rates in wild *Sus scrofa* populations are not well-understood.

In Japan, although CSF elimination was once declared in the domestic pig population and the OIE recognised Japan as a CSF-free country in 2015, the disease re-emerged in 2018 (13, 14) and Japan lost its disease-free status by 2020 (15). In addition to the absence of CSF for 26 years before the re-emergence, the clade-level genetic differences between the CSFV strain isolated from the index case of the outbreak in Japan in 2018 and the strains in the past outbreaks in Japan strongly suggested the re-introduction of CSFV from abroad (14). A phylogenetic analysis of CSFV suggested that the introduction occurred in April 2018, shortly before the detection of the first case (16). The first case of re-emerged CSF was found on a pig farm on 9th September 2018 and subsequent sporadic outbreaks in pig farms have been reported up to the present (15th February 2021) (17). After the confirmation of the first pig case, active surveillance for the wild boar captured and found dead in the area within 10 km of the infected pig farm was implemented in accordance with the national Guideline to Control Classical Swine Fever (17). Unfortunately, the CSFV entered wild boar populations also. The first CSFV infected wild boar was reported on 13th September 2018 and the epidemic that followed resulted in 3,166 PCR-positive cases of CSF as of 24th February 2021 (17). As of February 2021, the pig industry still suffers from the damage caused by CSF epidemics in wild boar populations as the transmission of CSF from the wild boar population resulted in economic loss due to the stamping-out of infected farms (18). The bait vaccination to wild boar since March 2019 and the preventive vaccination for domestic pigs since October 2019 using the live CSF-vaccine have been implemented around the epidemic areas (18). However, the control of the disease in wild boar populations and the prevention of transmission through domestic pigs-wild boar interface is recognised as an imperative task (19, 20).

Understanding the mechanism of disease dynamics with a modelling approach is important to plan evidence-based control measures against the epidemics of infectious diseases and for decision-making (21). In the case of CSF outbreaks, a modelling study to elucidate the mechanism of transmission in host populations has been attempted, which has contributed to the control program (22–24). To capture the disease dynamics, the estimation of epidemiological parameters, such as transmission rate ([the number of newly infected animals per unit time]/([the number of susceptible animals]^*^[the number of infected animals])), recovery rate ([the number of recovery per unit time]/[the number of infected animals]), and lethality rate ([the number of death due to infection]/[the total infected wild boar-time]), are essential. However, the parameter values are considered to vary largely between the strains of CSFV (8, 25). A low CFR has been reported for CSFV strain isolated from the outbreak in Japan in 2018; the experimental inoculation of the CSFV strain detected from domestic pigs in Japan (CSFV JPN/27/2019) in three naïve 8-week-old wild boar-pig hybrids and three naïve 8-week-old pigs resulted in the survival of 1 out of 3 hybrids and 3 out of 3 domestic pigs 29 days post-inoculation, respectively (i.e., 66.6% death in hybrids and 0% in pigs) (26). In addition, a field observation of a wild boar population in the CSF epidemic area reported only a limited reduction in population density (27). These observations regarding the CSFV strain in Japan suggest its relatively low CFR compared to those reported from the CSFV strains in other countries (2, 11, 21).

In addition to the variability of parameters, the lack and/or the sparsity of observation adds another difficulty for modelling, especially when targeting wildlife (28, 29). Except for transmission experiments with controlled settings (30–32), the estimation of key parameters for transmission dynamics has mostly been difficult because of the lack of time-series data on population density before and after the introduction of CSF. Furthermore, the sampling of wild animals is often complicated due to hunters' activity (29) and animal behaviour (33).

The present study aimed to estimate the two key parameters for estimating the dynamics of CSF in wild boar: the lethality rate and the recovery rate. To estimate them, detailed data on the population dynamics of wild boar are required; however, reliable estimation of the population size for wild boar is often challenging and lacks a standard methodological framework (34, 35). In this paper, we propose a method for estimating the lethality and recovery rates of CSF when detailed data on the population dynamics of wild boar are not available. In addition, using the estimates of the lethality and recovery rates, we also aimed to estimate CFR of CSF in the wild boar population in Japan.

## Materials and Methods

We propose a method for estimating the lethality and recovery rates of CSF without using detailed data on the population dynamics of wild boar, assuming (i) random sampling or full-observation of captured wild boar and (ii) equality of sampling pressure among infected individuals, recovered individuals, and others. Subsequently, we estimated the lethality rate and the recovery rate using field data on CSF epidemics obtained from September 2018 to March 2019 in the wild boar population of Gifu Prefecture, Japan.

### Conceptual Framework

#### Estimation

The most straightforward way to estimate CFR from CSFV infection is (i) following-up the infected wild boar, (ii) counting the number of death cases among them, and (iii) calculate the frequency of death among them (i.e., [the total number of dead animals by CSF]/[the total number of infected animals with CSF]). However, such data are difficult to obtain because of the cost and time required and the bias due to wildlife study. Sampling surveys are easier to conduct compared to the following-up survey of the infected wild boar. Another way to estimate the CFR is to estimate from the time-series data of infected and dead wild boar using a mathematical model describing the transmission process of infectious diseases (e.g., Susceptible-Exposed-Infectious-Recovered [SEIR] model) (21, 28).

Estimations of the epidemiological parameters (e.g., transmission coefficient, recovery rate, and lethality rate) using the SEIR model have been conducted frequently. It has also been applied to the dynamics of CSF in wild boar [e.g., Stahnke et al. (29)]. However, they require spatial heterogeneity of contact between hosts, which are difficult to quantify in the epidemiological studies of wildlife. From the sampling survey used in our study, the following data were obtained: (i) the number of sampled wild boar (ii), the number of infected wild boar, determined by a conventional reverse transcription polymerase chain reaction (RT-PCR) test, and (iii) the number of recovered wild boar, determined by RT-PCR and enzyme-linked immunosorbent assay (ELISA) tests. To estimate the CFR from CSF using only these three data points, we constructed a mathematical model describing the transmission and the natural history of CSF among wild boar (i.e., the disease progression process of CSF in wild boar at individual level over time) and derived the relationship between the CFR and these three data points.

#### Mathematical Model of CSF

Based on previous modelling studies of CSF in wild boar populations (21, 29) and feral pigs (28, 36), the transmission dynamics of CSF can be written as follows:


(1) $$\begin{array}{l} \frac{dS_{i}}{dt}=b\;(t)\;N_{i}\;(t)-S_{i}\;(t)\;\sum_{j}\left(\beta_{i,j}I_{j}\;(t)\right)-\mu S_{i}\;(t), \end{array}$$

(2) $$\begin{array}{l} \frac{dE_{i}}{dt}=S_{i}\;(t)\;\sum_{j}\left(\beta_{i,j}I_{j}\;(t)\right)-(\mu +\varepsilon )\;E_{i}\;(t), \end{array}$$

(3) $$\begin{array}{l} \frac{dI_{i}}{dt}=\varepsilon E_{i}\;(t)-(\mu +\gamma +\mu_{d})\;I_{i}\;(t), \end{array}$$

(4) $$\begin{array}{l} \frac{dR_{i}}{dt}=\gamma I_{i}\;(t)-\mu R_{i}\;(t), \end{array}$$

(5) $$\begin{array}{l} \frac{dD_{i}}{dt}=\mu \left(S_{i}\;(t)+E_{i}\;(t)+I_{i}\;(t)+R_{i}\;(t)\right)+\mu_{d}I_{i}\;(t), \end{array}$$

(6) $$\begin{array}{l} N_{i}\;(t)=S_{i}\;(t)+E_{i}\;(t)+I_{i}\;(t)+R_{i}\;(t). \end{array}$$

where *S*_*i*_(*t*), *E*_*i*_(*t*), *I*_*i*_(*t*), *R*_*i*_(*t*), and *D*_*i*_(*t*) denote the number of susceptible, latent, infectious, recovered, and dead wild boar in spatial unit *i* at time *t*, respectively. *N*_*i*_(*t*) denotes the total number of surviving wild boar in spatial unit *i* at time *t*. β_*i,j*_, ε, γ, *b*, μ, and μ_*d*_ represent the transmission coefficient from spatial unit *j* to *i*, the progression rate from latent to infectious, the recovery rate, the reproduction rate, the natural mortality rate (the cause of death other than CSF), and the lethality rate of CSF, respectively. From the model above, the number of wild boar at time *t, N*_*i*_ (*t*), during the CSF epidemic can be written as


(7) $$\begin{array}{l} \frac{dN_{i}}{dt}=(b-\mu )\;N_{i}\;(t)-\mu_{d}I_{i}\;(t). \end{array}$$

#### Estimation of CFR Using the Mathematical Model

Introducing a new variable describing time, τ, and solving equation (7), we have:


(8) $$\begin{array}{l} N_{i}\;(t)=N_{i}\left(0\right)\exp\left(\left(b-\mu \right)\;t-\mu_{d}\int_{\tau =0}^{t}\frac{I_{i}\;(\tau )}{N_{i}\;(\tau )}d\tau \right). \end{array}$$

The survey period in this study (September 2018-March 2019) did not cover the main reproduction period of wild boar in Japan, which is spring (April-May) (37). Therefore, we assumed no reproduction of wild boar during the survey period, that is, *b* = 0. Assuming a constant sampling probability σ over time among spatial units, regardless of the infection status, the expected total sampled numbers, ${\hat{N}}_{i}\left(t\right)$, the number of sampled and infected wild boar, ${\hat{I}}_{i}\left(t\right)$, and the number of sampled and recovered wild boar, ${\hat{R}}_{i}\left(t\right)$, were


(9) $$\begin{array}{l} {\hat{N}}_{i}\;(t)=\sigma N_{i}\;(t), \end{array}$$

(10) $$\begin{array}{l} {\hat{I}}_{i}\;(t)=\sigma I_{i}\;(t), \end{array}$$

(11) $$\begin{array}{l} {\hat{R}}_{i}\;(t)=\sigma R_{i}\;(t), \end{array}$$

From Equations (8), (9), and (10), the relation between ${\hat{N}}_{i}\left(t\right)$ and μ_d_ can be written as


(12) $$\begin{array}{l} {\hat{N}}_{i}\;(t)={\hat{N}}_{i}\left(0\right)\exp\left(-\mu t-\mu_{d}\int_{\tau =0}^{t}\frac{{\hat{I}}_{i}\left(\tau \right)}{{\hat{N}}_{i}\left(\tau \right)}d\tau \right). \end{array}$$

From Equation (12), the value of μ_d_ can be calculated if the values of ${\hat{N}}_{i}\left(0\right)$, ${\hat{N}}_{i}\left(t\right)$, ${\hat{I}}_{i}\left(t\right)$ and μ are given. ${\hat{N}}_{i}\left(0\right)$, ${\hat{N}}_{i}\left(t\right)$, ${\hat{I}}_{i}\left(t\right)$ are given by the data, and μ is assumed to be 0.15 per year according to a previous study (38), then the value of μ_d_ can be calculated. Also, from Equations (4), (9), (10), and (11), the relationship between the number of recovered wild boar, ${\hat{R}}_{i}\left(t\right)$, and γ can be written as


(13) $$\begin{array}{l} {\hat{R}}_{i}\;(t)={\hat{N}}_{i}\;(t)\left(\gamma \int_{\tau =0}^{t}\frac{{\hat{I}}_{i}\left(\tau \right)}{{\hat{N}}_{i}\left(\tau \right)}d\tau -\mu \int_{\tau =0}^{t}\frac{{\hat{R}}_{i}\left(\tau \right)}{{\hat{N}}_{i}\left(\tau \right)}d\tau \right). \end{array}$$

Similar to μ_d_, the value of γ can be calculated from Equation (13). For the estimation of μ_d_, using Equation (12), the likelihood function describing the Poisson sampling process of wild boar is defined as


(14) $$\begin{array}{l} \underset{t}{\prod }\text{pmf }\left(\text{Poisson }\left({\hat{N}}_{i}\;(t)\right),{\hat{N}}_{data,i,t}\right), \end{array}$$

where pmf(Poisson(λ), *x*) denotes the probability mass function of the Poisson distribution with the expected value = λ conditioned on observation = *x*, and ${\hat{N}}_{data,i,t}$ represents the time-series data of the number of sampled wild boar in spatial unit *i* at time *t*, respectively. For the estimation of γ, using equation (13), the likelihood function describing the binomial sampling process of recovered wild boar was


(15) $$\begin{array}{l} \underset{t}{\prod }\text{pmf }\left(\text{Bin }\left({\hat{N}}_{data,i,t},{\hat{R}}_{i}\;(t)/{\hat{N}}_{i}\;(t)\right),{\hat{R}}_{data,i,t}\right), \end{array}$$

where pmf(Bin(*n, p*), *x*) denotes the probability mass function of the binomial distribution with the trial number = *n* and probability *p* conditioned on observation = *x*, and ${\hat{R}}_{data,i,t}$ represents the time-series data of the number of sampled and recovered wild boar in spatial unit *i* at time *t*, respectively. The likelihood functions, as shown in Equations (14) and (15), were maximised to estimate μ_d_ and γ. Using the estimates of μ_d_ and γ, CFR can be calculated as CFR = μ_d_/(γ+μ_d_+μ) (39). The 95% confidence intervals (CIs) for the estimates were calculated using bootstrap resampling. All computations were performed using Mathematica ver. 12.0.0.0 (62).

### Data of the CSF Epidemic in Wild Boar in Japan

The CSF epidemic in Japan emerged on 9th September 2018 in a pig farm located in Gifu City, Gifu Prefecture and gradually expanded to other cities (19, 20). In Gifu prefecture, no routine fine-scale surveillance for CSF in wild boar was conducted before the first report of CSF in domestic pigs. Shortly after the first report in domestic pigs, the Gifu Prefectural government began investigating CSF invasion in wild boar populations around the focus of the outbreak. In this active surveillance, all wild boars captured and found dead in the area within 10 km of the affected pig farms were tested for CSF. The first case of CSF in wild boar, which was detected from a dead individual found at 7.4 km away from the index pig farm (18), was reported on 13th September 2018 (17).

When an infected wild boar or domestic pig was reported as PCR positive, the Gifu Prefectural government designated the areas within a 10 km radius from the geographical points where PCR positive animals were found as the “intensive surveillance area” (18). Once an area was identified as an intensive surveillance area, only permitted investigators (mainly, voluntary hunters) and hunters working for nuisance control were allowed to capture wild boar; all private hunting activities were restricted. The sampled wild boar in the intensive surveillance area, comprising captured wild boar and found-dead wild boar, were examined for CSF by public veterinary health services using RT-PCR for the viral genome and ELISA for antibodies against CSFV. The sampling scheme of specimens and protocols for RT-PCR and ELISA tests have been described previously (40). If the samples were not appropriate for these tests, the examinations were not conducted. The date and geographical data (i.e., spatial coordinates) of the sampled wild boar were recorded. We obtained data from the Gifu Prefectural Government.

### Data Processing

We transformed the data to apply our modelling approach. We defined the mesh unit in the hunting mesh of Gifu Prefecture as a spatial unit for our analysis. The hunting mesh consists of 28 × 36 meshes located between 136° 7' 30” E and 137° 52' 30” E in longitude and between 35° 0' 0” N and 36° 34' 60” N in latitude, implying that the size of one mesh is about 4.6 × 5.6 km. The data on population size and density of wild boar were not available due to the lack of reliable records and estimation methods. Setting the first day as 13th September 2018 (the day when the first infected wild boar was found), the number of tested wild boar (captured wild boar and wild boar found dead), the PCR-positive wild boar regardless of the result of the ELISA test (infected wild boar), and PCR-negative and ELISA-positive wild boar (recovered wild boar) were totalled up for 7 days for each mesh. The individuals with PCR-negative but were not tested for ELISA were excluded from the analysis. The PCR-positive and ELISA-positive animals were included as infected animals because the possibility of viral transmission from them was not negligible. The monotonicity of time-series change (i.e., monotonic increase/decrease or not) in the trend of the number of sampled wild boar over sampling time points was tested by the Jonckheere-Terpstra Trend Test with a significance level of 0.05.

We selected the meshes that were covered by intensive surveillance areas designated on 25th September 2018, the first day of the designation. This enabled us to satisfy the assumption of a “full observation of captured or found dead wild boar” by restricting hunting activities (started in October 2018) other than for CSF surveillance. We used the data until 28th February 2019 (i.e., the last day of the 24th week from 13th September 2018) to avoid the influence of (i) the reduced sampling activity in early March and (ii) oral vaccination for wild boar that started on 26th March 2019. Data processing was performed using R version 4.0.2 (41) and QGIS version 3.16 (42).

## Results

### The Numbers of Sampled and Tested Wild Boar

**Figure 1:**
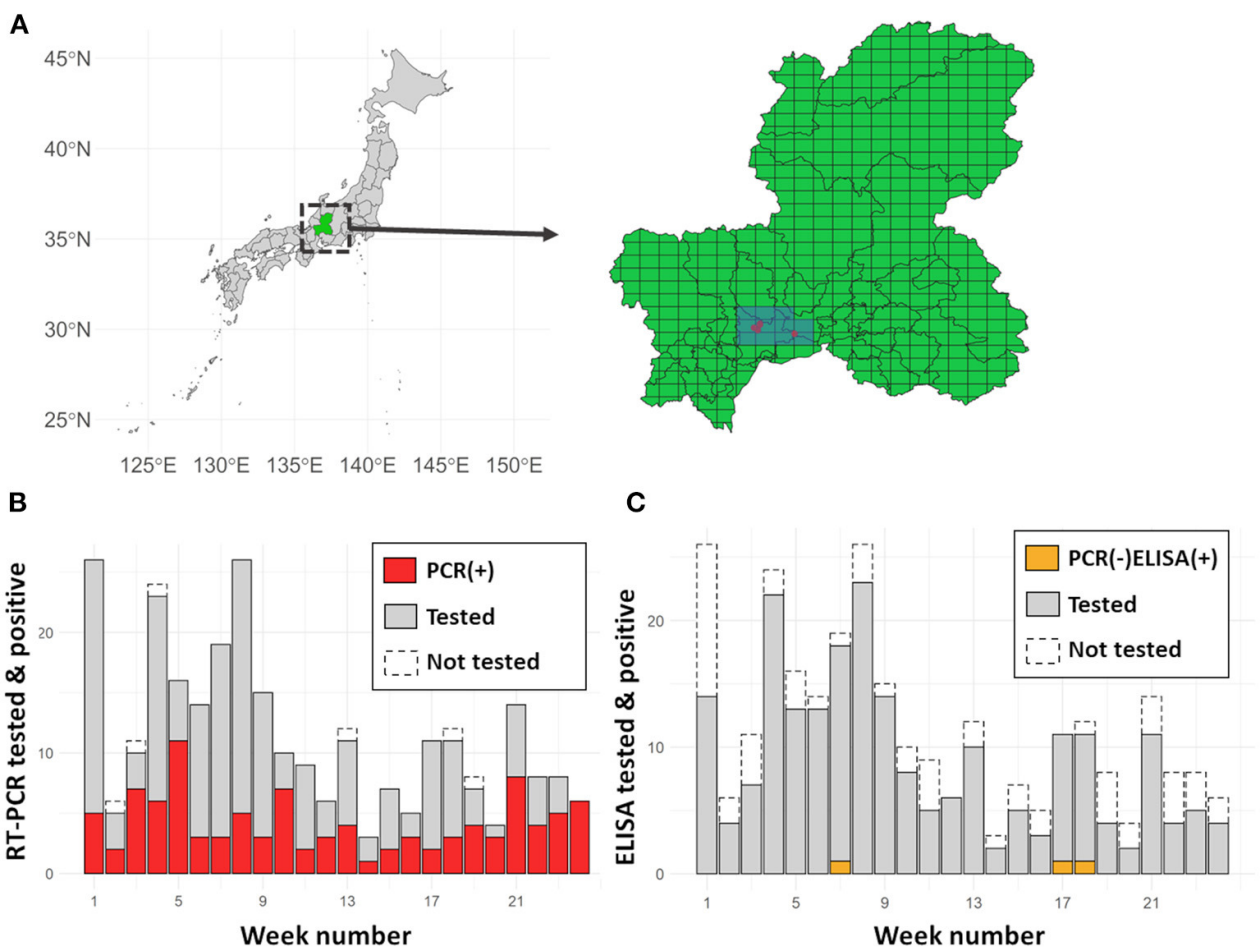
**(A)** The location of Gifu Prefecture in Japan and sampling meshes. Gifu prefecture (green) is located in central Japan. In the expanded figure, red dots denote the locations where PCR(+) wild boar were found before 25th September 2018. All the selected meshes (blue, 11 meshes) were included in the intensive surveillance area. **(B)** Number of wild boar tested weekly by RT-PCR vs. those who tested positive. The red and grey bars denote PCR(+) and PCR(–) wild boar in the investigated 24 weeks, respectively. The area framed with dashed lines shows the number of hunted but not tested individuals. **(C)** Number of wild boar tested weekly by ELISA vs. those who tested positive. The orange bar (at the bottom of week 7, 17, and 18) denotes ELISA-positive PCR-negative wild boar. The grey bar denotes ELISA-negative wild boar. As in the case of **(B)**, the area framed with dashed lines shows the hunted but not tested individuals.

Six RT-PCR positive [PCR(+)] wild boar were reported before 25th September, and 11 meshes covered the area within a ten-kilometre radius from the place where the PCR(+) wild boar were found (Figure 1). Among these meshes, the weekly number of sampled wild boar ranged from 3 (week 14) to 26 (weeks 1 and 8), and 280 wild boar were sampled in total. The number of sampled wild boar showed a decreasing trend over time (Jonckheere-Terpstra Trend Test, *p* < 0.001). Among the sampled wild boar, 274 and 219 were tested using RT-PCR and ELISA, respectively. The number of PCR(+) wild boar per week ranged from 1 (week 14) to 11 (week 5), and a total of 102 wild boar (37.2% of all the RT-PCR-tested individuals) were recorded as PCR(+). The number of wild boar that were RT-PCR-negative [PCR(–)] and ELISA positive [ELISA(+)] was quite small; 3 out of 219 (1.4%) individuals, one each found in weeks 7, 17, and 18.

The proportion of PCR(+) individuals showed a significant increasing trend over time (*p* = 0.02, by the Jonckheere-Terpstra Trend Test), whereas PCR(–) and ELISA(+) wild boar did not show a significant change in the trend (p = 0.39, by the Jonckheere-Terpstra Trend Test).

### The Estimation of Lethality Rate, Recovery Rate, and Case Fatality Ratio

**Figure 2:**
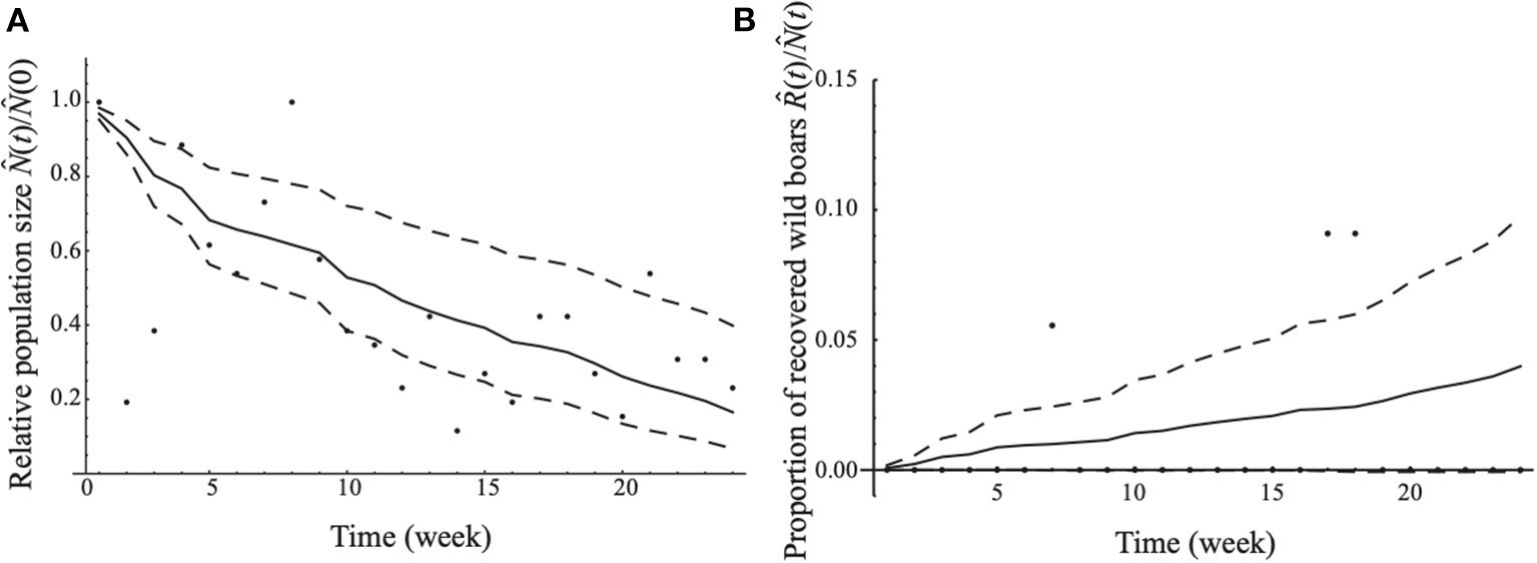
The model fitting with the data. The median estimated relative population size [in **(A)**] and the median estimated proportion of recovered wild boar [in **(B)**] are demonstrated as solid lines. The 95% confidence intervals are denoted by dashed lines. Each dot is the observed value of the relative population size [in **(A)**] and proportion of recovered wild boar [in **(B)**] in each week.

The estimated values of the lethality rate and recovery rate of CSF were 0.165 per week (95% CI: 0.081–0.250) and 0.004 per week (95% CI: 0–0.009), respectively. A model fit with the data using our estimate with respect to the time series change in the relative population size between week *t* and week 1 (i.e., $\frac{{\hat{N}}_{i}\left(t\right)}{{\hat{N}}_{i}\left(0\right)}$) and that in the proportion of recovered wild boar ($\frac{{\hat{R}}_{i}\left(t\right)}{{\hat{N}}_{i}\left(t\right)}$) is shown in Figure 2. According to the estimate of the lethality rate, recovery rate, and natural mortality rate, the case fatality ratio was estimated to be 0.959 (95% CI: 0.904–0.981).

### Sensitivity Analysis of CFR

To test the robustness of the CFR, we conducted two sensitivity analyses: the sensitivity of CFR against the change in (i) natural mortality, and (ii) recovery rate. Since natural mortality can differ according to the environment [e.g., the amount of food resources, population density, existence of predators, climate, etc. (43)], we assumed the possible range of the annual mortality rate to be 0.10–0.50. Our estimate of the recovery rate differed from that reported previously (21). We also conducted a sensitivity analysis for the estimated CFRs with the change in recovery rate ranging around our estimate; from 0.05 to 0.25 per year (0.001–0.006 per week).

**Figure 3:**
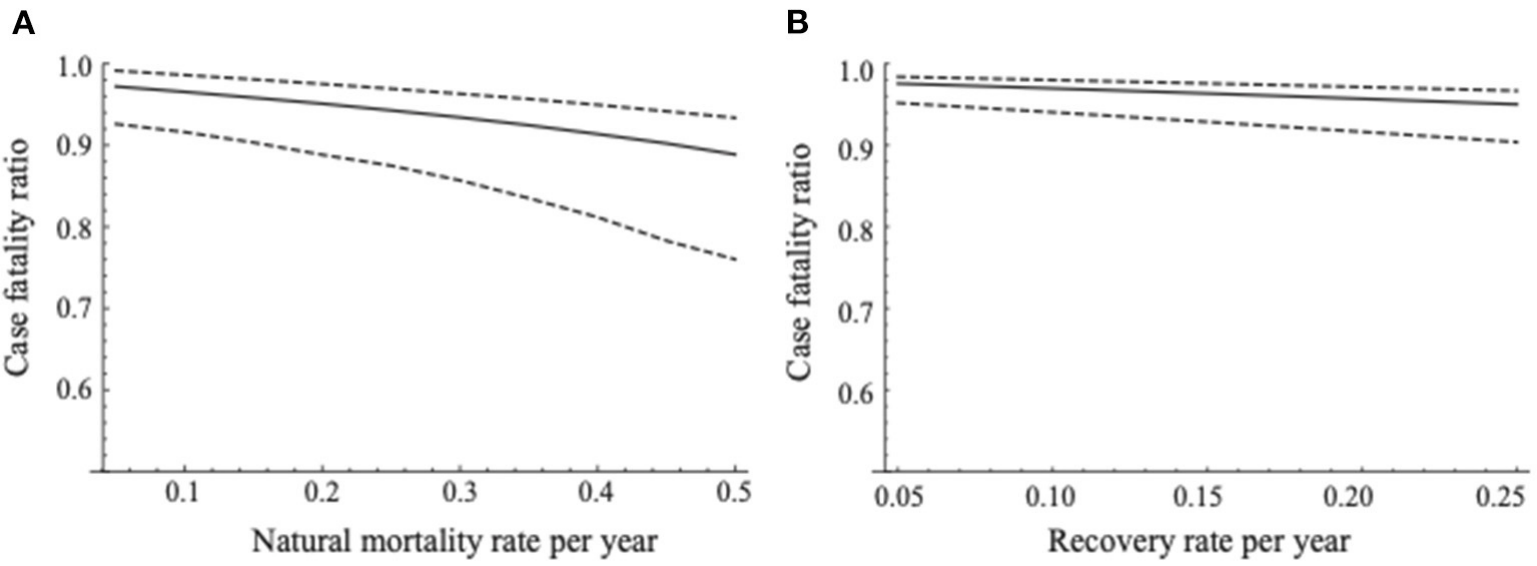
Sensitivity of the case fatality ratio with the estimated recovery rate in the present study. **(A)** Represents sensitivity of the case fatality ratio against the natural mortality rate and **(B)** represents that against the estimated recovery rate in the present study. The changes in the median estimated CFR are demonstrated as the solid line. The dashed lines denote the 95% confidence intervals of estimated CFRs.

The sensitivity of the CFR against the natural mortality rate (μ) is shown in Figure 3A. As the natural mortality rate increased, the estimates of CFR tended to decrease with wider confidence intervals. However, the estimate of CFR did not change largely; it ranged between 0.889 and 0.972.

The sensitivity of the CFR against the recovery rate is shown in Figure 3B. As with the change in the natural mortality rate, the estimates of the CFR gradually decreased with wider confidence intervals by increasing the recovery rate. The estimates of CFR did not vary largely, ranging between 0.950 and 0.976.

## Discussion

The present study estimated the lethality and recovery rates of the CSF in the wild boar population in Japan, without using detailed data on the population dynamics of wild boar. Considering the duration between the infection and detection of viral gene (e.g., 3–5 days post infection [i.e., dpi) with CSFV in wild boar-pig hybrids (26)] and between the infection and the development of antibody-mediated immunity [e.g., 10–12 dpi in wild boar-pig hybrids (26)], our investigation period made some cycles of CSF transmission in wild boar observable. Our results show that CSF in Japan shows similar lethality rate and lower recovery rate compared to the estimates for European countries (21). Utilising the estimated values, the CFR of CSF in the wild boar population was also estimated. Our estimate of CFR is close to that reported by Artois et al. (2).

To the best of our knowledge, the present study is the first study which estimated the lethality rate of CSF and recovery rate from CSF at the same time from the observed CSF epidemiological data in wild *Sus scrofa* populations. To estimate them, we construct a mathematical model of CSF epidemics among wild boar and derived the relation between the lethality rate of CSF, recovery rate from CSF, and the epidemiological data, i.e., the time-series data of the number of infected, recovered, and captured wild boar. The disease dynamics of CSF in wild boar and feral pig populations have been studied using mathematical modelling in Pakistan (36), France (21), Germany (29), Australia (28), and hypothetical populations (44, 45). In the context of the infectious disease epidemic in wild boar, there are also many modelling studies regarding African swine fever (46–53). However, most mathematical modelling studies have focused on detailed spatial and/or temporal dynamics of the disease and aimed at either the estimation of transmissibility or the simulation of transmission dynamics in the population.

The lethality and recovery rates of CSF have often been estimated from transmission experiments in a limited setting, though the actual morbidity and lethality of CSF show heterogeneity due to the differences in the viral strain, host taxonomy, and living environment of the virus/host (2). The current strain of CSFV in Japan belongs to sub-genotype 2.1d (54). Sub-genotype 2.1d of CSFV has been widespread mainly in East Asia, reporting moderate lethality in domestic pigs compared to the highly virulent strain in Europe (18). The estimation method proposed in the present study can determine the heterogeneity of the morbidity and lethality of CSF, even if the transmission experiment is not conducted.

The lethality rate from CSF estimated in the present study, 0.165 per week, falls within the range of mortalities reported previously, although only a few previous studies considered the lethality rate of CSF in wild boar, which ranged from 0.021 per week (5% of infected wild boar die in 15 days) to 0.562 per week (~70% of infected wild boar die in 15 days). In this range, the lower limit is for adult wild boar [assumed by the Panel on Animal Health and Welfare of European Food Safety Authority (21)] and the upper limit is for piglets [confirmed in an experimental transmission of highly virulent strain (21)]. The data used in this study came from a mixed population of adults and piglets (Gifu Prefectural Government, personal communication, 4 November 2020) although detailed data on the age of sampled wild boar is not available in the present study. Our estimate of the lethality of CSF agrees with the estimates reported previously.

In contrast to the lethality of CSF, the estimated value of the recovery rate from the observed data in Gifu Prefecture (0.004 per week) was lower than those previously reported [previously reported/assumed recovery rates ranged from 0.01 per week (29) to 0.416 per week (21)]. The possible reasons why we obtained the low value can be explained by two factors: (i) the nature of CSFV strain in Japan or Japanese wild boar, and (ii) the stochastic effect in the small sample size. Regarding (i), infection period of CSFV among wild boar in Japan is longer than that of previously reported in other countries due to the strain of CSFV in Japan or Japanese wild boar. As for the CSFV in Japan (i.e., CSFV JPN/27/2019), the virulence is considered to be moderate (18, 26, 55). There is a possibility that infected wild boar in Japan may show mild symptoms and long infection period as seen in the case of a moderate strain [e.g., ≥70 days long lasting infection by a moderate CSFV strain reported by Donahue et al. (56)]. Indeed, we observed some wild boar maintained the PCR(+) status even antibody was produced; the number of PCR(+) and ELISA(+) boar was 17 among 219 tested wild boar (7.8%), which is larger than the number of PCR(-) and ELISA(+), 3 (1.4%) (Fisher's exact test, *p* = 0.002). This implies that the wild boar might be a career of CSFV with maintaining the PCR(+) status. If the recovery events, which occur at the timing of the end of infection period, were censored in the week 24 (i.e., the end of the observation period in the present study), it might result in an underestimation of the recovery rate. As for (ii), there is a possibility that the recovery event, which occurs after an infection period, was too rare to be detected in the limited sample size and might result in a lack of observation. A systematic survey to sample a sufficient number of wild boar is important for understanding the current situation of CSF.

The CFR estimated in the present study depends on the estimates of the lethality rate of CSF, natural mortality rate, and recovery rate. Hence, the CFR should be calculated using appropriately estimated values and ranges of these parameters. Indeed, the estimate of CFR calculated by a recovery rate based on our observed data showed a high value: 0.959 (95% CI: 0.904–0.981), while that calculated by a recovery rate based on a previous study [0.416 per week, calculated from 1/γ = 13 days (21)] showed a lower value: 0.282 (95%CI: 0.161–0.375). The accuracy of the recovery and natural mortality rates should be secured to estimate the CFR.

The estimates of epidemiological parameters and the CFR in the present study will not be extrapolated simply to the CSF prevailing in other areas. If the CSF is caused by a different strain of CSFV from the present study, the characteristics of the different CSFV strain (e.g., virulence and pathogenicity) can influence the estimates of the parameters and the CFR. Also, the estimates of the parameters and the CFR can be influenced by the characteristics of a wild boar population (e.g., susceptibility and age structure). Rather than extrapolating the estimates of the present study, our methodology is applicable for the CSF with those different CSFV strains and/or host population settings. The epidemiological parameters obtained by our methodology can be utilised for the prediction/simulation of CSF epidemics, planning of intervention scheme, and further evaluation of the effect of intervention in the area.

Estimates of recovery rate is a key for calculation of CFR, however, accurate estimation of the recovery rate by our modelling approach is difficult in Japan due to the short research period. The distribution of oral vaccination had started at the end of March 2019, and there was no room to apply our estimation method. Therefore, evaluation by a transmission experiment using wild boar was the most realistic solution for estimating the recovery rate. However, the estimation by the transmission experiment may be difficult to conduct for moderately virulent strains due to possible long term infection. If long-term infection occurs with the moderately-virulent strain of CSFV in Japan, a sufficiently long experimental period is required. Considering the cost of the long-term experiment, capacity building will also be required.

Estimation of the natural mortality rate is also difficult. The mortality of wild animals is often estimated by analysing the survival data of animals obtained from the capture-mark-recapture method (38, 57), the radio tracking method (63), and the change in their age structure and population dynamics (58). However, in Japan, data on marking/radio-tracking and population dynamics are limited in most areas of CSF epidemics. Furthermore, since the mortality of wild boar is also influenced by the intensity of hunting (38) and environmental conditions (59), the natural mortality rate may vary, depending on the area and time. The estimation of natural mortality should be conducted with the detailed data of the wild boar population, hunting activity, and environment for the wild boar in terms of sampling time interval and sample size for each area.

In addition to the limitations in the estimation mentioned above, a few limitations of the present study should be noted. Firstly, we could not explicitly consider the sampling pressure in our model. A precise estimate of population size is required to take the detailed sampling pressure into account; however, it has been difficult to estimate the population of wild boar, and the methodology for estimation has long been discussed (34). Secondly, we simplified the heterogeneity of sampling probabilities between different health states of wild boar (i.e., an equal sampling probability in any state of *S*, *E*, *I*, and *R*). If infected individuals lose their activity and are difficult to sample compared to other statuses, or are more easily found as dead individuals, they violate the equality of sampling rate. The proportion of PCR(+) in the found dead wild boar (92.9%: 39 in the 42 RT-PCR-tested individuals) and that in the captured wild boar (27.2%: 63 in the 232 RT-PCR-tested individuals) were significantly different (χ^2^-test, *p* < 0.001). The difference in the proportion of PCR-positive between found dead wild boar and captured wild boar suggested that the discovery rate of found dead wild boar may influence the estimate of morality rate. The behaviour and discovery process of wild boar infected with CSF needs to be studied further in the future. Thirdly, although we conducted a sensitivity analysis for natural mortality (μ) assuming a change in mortality other than CSF, we did not clearly incorporate the impact of capturing, i.e., the loss of population density, in the 24 weeks of the investigation period. The impact of capturing can only be clarified when the population estimates of wild boar are obtained, however, it is not available so far. Once it is available, the relationship between capturing intensity and population density of wild boar should be integrated to estimate the lethality rate of CSF. Fourthly, we did not take the movement of wild boar in and out of the investigated area into account. This is because wild boar is generally considered to be sedentary with accepting the overlap of their home-ranges between herds (37); they usually move within a small home-range [1.0–8.0 km^2^ on average (60)] compared to the investigated area of the present study (283.4 km^2^). Podgórski et al. (61) also reported that wild boar only sporadically beyond the 3 km distance. While we assumed the sporadic long-distance movement of wild boar will be negligible, the frequency of such events has not been measured in Japan. The movement and the contact rates in Japanese wild boar should be clarified and carefully considered in future. Finally, we did not include the sensitivity and specificity of RT-PCR and ELISA in our model. This is because the sensitivity and specificity of CSFV are generally very high [about 99% sensitivity and specificity in RT-PCR and about 99% sensitivity and specificity in ELISA (21)]. However, there is a lack of accurate information on the test sensitivity and specificity of the CSFV JPN/27/2019 strain circulating in Japan. This should be taken into account in the future, together with a consideration of the condition of specimens in field settings.

Despite these limitations, the present study developed a method to estimate the lethality rate of CSF and the recovery rate of CSF in wild boar populations without using the results of transmission experiments. This method is useful for understanding the dynamics of CSF through the lethality rate of CSF and the recovery rate of CSF. The estimates of those epidemiological parameters without the influence of the bait vaccination were obtained in the present study. The estimates will play a key role for further evaluating the effectiveness of bait vaccine for wild boar and also for development of the optimal vaccination scheme in Japan. The estimate of the recovery rate in the Japanese wild boar population was lower than that in previous studies; it can be hypothesised that the moderately virulent CSFV in Japan is likely to cause a longer course of infection. It can also induce an underestimation of the recovery rate by censoring recovery events due to short survey period. For the accurate estimation of CFR in wild boar, the implementation of a long-term transmission experiment is required to elucidate the average recovery rate and its variance.

## Data Availability Statement

The original contributions presented in the study are included in the article, further inquiries can be directed to the corresponding author.

## Author Contributions

RO conceived the study and developed the mathematical model. RM, YH, and TY collected the data. RM cleaned the data and conducted the spatial analysis. The computation was performed by RO. RO and RM contributed to the interpretation of the results and drafting of the early version of the manuscript. YH and TY further revised the manuscript. All authors gave comments on the revised manuscript and approved the final version of that.

## Funding

This project was conducted under the research project on regulatory research projects for food safety, animal health, and plant protection (JPJ008617.20319390) funded by the Ministry of Agriculture, Forestry and Fisheries of Japan. In addition, RM received funding supports from JSPS KAKENHI (Grant Number 19KK0242) and RO was supported by CREST (Grant Number JPMJCR1413) from Japan Science and Technology Agency (http://www.jst.go.jp/). The funders had no roles in the study design, data collection and analysis, decision to publish, or manuscript preparation.

## Conflict of Interest

The authors declare that the research was conducted in the absence of any commercial or financial relationships that could be construed as a potential conflict of interest.

## Publisher's Note

All claims expressed in this article are solely those of the authors and do not necessarily represent those of their affiliated organizations, or those of the publisher, the editors and the reviewers. Any product that may be evaluated in this article, or claim that may be made by its manufacturer, is not guaranteed or endorsed by the publisher.

## Abbreviations

CSF: classical swine fever
CFR: case fatality ratio
RT-PCR: reverse transcription polymerase chain reaction
ELISA: enzyme-linked immunosorbent assay
CI: confidence interval
CSFV: CSF virus
EFSA: European Food Safety Authority.
